# Testing the ELSA Birth App During Pregnancy and Labor for Primiparous Women: Randomized Controlled Trial

**DOI:** 10.2196/72807

**Published:** 2025-10-16

**Authors:** Karin Ängeby, Margareta Johansson, Elin Børøsund, Cecilie Varsi, Leonardo Horn Iwaya, Anna Nordin

**Affiliations:** 1 Department of Health Science, Faculty of Health, Science, and Technology, Karlstad University, Universitetsgatan 2, Karlstad, 65188, Sweden, 46 703579767; 2 Women's Department and Centre for Clinical Research and Education, Region Värmland, Karlstad, Sweden; 3 Department of Women’s and Children’s Health, Uppsala University, Uppsala, Sweden; 4 Department of Women’s Health Care, Akademiska University Hospital, Uppsala, Sweden; 5 Department of Digital Health Research, Division of Medicine, Oslo University Hospital, Oslo, Norway; 6 Department of Nursing and Health Sciences, Faculty of Health and Social Sciences, University of South-Eastern Norway, Drammen, Norway; 7 Faculty of Health and Social Sciences, University of South-Eastern Norway, Drammen, Norway; 8 Department of Mathematics and Computer Science, Faculty of Health, Science, and Technology, Karlstad University, Karlstad, Sweden

**Keywords:** fear of childbirth, mobile app, early labor, emotional distress, midwifery support, antenatal education, mHealth, childbirth experience, randomized controlled trial, mobile health

## Abstract

**Background:**

Early labor is often managed at home without professional support. The Birth App (Birth by Heart) is an app designed to support women during early labor. A pilot study revealed that women found the app’s exercises simple, understandable, and practical. The app was perceived as useful and appreciated by women, although areas for improvement were identified, primarily related to technical issues. After the development and test period, the updated app was tested in a randomized controlled trial.

**Objective:**

This study aims to investigate whether women using the Birth App during pregnancy and childbirth experience less distress during early labor compared to those receiving standard antenatal care.

**Methods:**

We used online recruiting in a nonblinded 3-part blended care model with 1:1:1 randomization: group 1 (Birth App intervention); group 2 (Birth App Plus, combining the app with in-person additional midwifery contacts); and group 3 (control group receiving standard antenatal care). Pregnant nulliparous women were invited via social media. Eligibility criteria were nulliparity, planning a vaginal birth, from gestational week 25+0 to 35+6 weeks, proficiency in understanding Swedish, and having access to a smartphone or tablet. Data were analyzed with descriptive statistics, chi-square tests, and ANOVA.

**Results:**

A total of 391 women completed the baseline questionnaire and were included in the study (group 1, n=118; group 2, n=114; group 3, n=118). Of these, 335 women responded to the questionnaire 1 month postpartum, yielding a response rate of 85.6%. Most participants experienced a spontaneous onset of labor (group 1: 67/103, 65%; group 2: 81/114, 71%; and group 3: control group, 86/118, 73%), with no statistically significant differences between groups. During early labor, women in group 1 remained at home for a mean of 16.76 (SD 20.45) hours, group 2 for a mean of 14.47 (SD 16.82) hours, and the control group for a mean of 12.90 (SD 15.99) hours (*P*=.32). For the primary and secondary outcomes, only women with spontaneous onset of labor (n=234) were included in the analysis. The primary outcome, emotional distress, showed similar mean values across all groups. No statistically significant differences were identified in the secondary outcomes: childbirth experience, pain relief, and support from the partner. However, for the secondary outcome fear of future birth, a pairwise testing from baseline to follow-up revealed a statistically significant mean difference for the intervention groups (group 1: mean 13.53, 95% CI 5.12‐21.92, *P*=.002; group 2: mean 14.59, 95% CI 7.75‐21.42, *P*<.001) with a medium effect size (Cohen *d*=.40 vs *d*=.47). For group 3, the mean was 6.78 (95% CI −.95 to 14.53; *P*=.08).

**Conclusions:**

The Birth App, in conjunction with additional midwifery support, can be a valuable tool for pregnant women and their partners during pregnancy and childbirth. The observed reduction in fear of forthcoming childbirth associated with the Birth App warrants further investigation.

## Introduction

The first part of childbirth, called early labor, is often handled at home by the women and their partners themselves, without support from professionals [1]. However, it could be difficult for first-time mothers to know what to expect at labor onset, and their experiences in early labor may differ from their expectations [2]. A newly published scoping review showed that positive and negative emotions were perceived during early labor [2]. The Early Labor Experience Questionnaire (ELEQ) was developed to assess women’s affective experiences [3], showing primiparous women experiencing higher emotional distress during early labor compared to multiparous women [4]. Emotional distress during childbirth, perceived lower quality of care, and a negative childbirth experience in general are associated with a longer latent phase of labor in first-time mothers [5]. However, women are encouraged by professionals not to be in the labor ward during early labor, to reduce the risk of the use of unnecessary medical interventions [6].

Structured antenatal education programs to prepare for childbirth are offered to women and their partners, and they address both physiological, emotional, and social aspects during pregnancy, childbirth, and early parenthood. However, these programs vary across countries and contexts, and the benefits of these programs remain unclear [7]. Several studies have shown that special targeted antenatal education enabling the woman and her partner to feel more confident is beneficial for the outcome of labor, both in terms of arriving at the hospital in more advanced labor, less use of epidural anesthesia, a better childbirth experience, and fewer acute cesarean sections [8-10].

To enhance women’s confidence to remain at home during early labor, adequate preparation for managing pain in early labor is important [1], as feeling anxious increases the perception of pain [2]. Birth preparation training for women, with mindfulness, has been tested in a few small, randomized controlled trials (RCTs), demonstrating a higher ability to manage pain, increased psychological well-being, a trend toward lower use of opioids during labor, increased self-efficacy, and decreased fear of childbirth among women [11,12].

Since 2009, pregnant women and their partners have had the option to enroll in antenatal training classes, for a fee, using the Birth Without Fear method [13]. This method aims to equip women and their partners with the knowledge and skills to better manage pain and experiences during childbirth through active measures, including support and stress-relief techniques. The method uses 4 different tools to enhance relaxation: soft and silent breathing through contractions, relaxed muscles, a deep pitch of the voice, and positive expectation of the mind [13]. The Birth Without Fear method was later conceptualized in a digital app, and a first version was tested in a feasibility pilot study. The result showed that the app was perceived as useful and appreciated by women and suitable for efficacy testing in an RCT [14]. The company Birth by Heart led the app development, involving senior software developers with expertise in health and fitness apps and an expert group to define the requirements. Regular stakeholder meetings ensured alignment, reviewed app versions, and incorporated feedback throughout the process. The app development process is more thoroughly described in another publication [15]. In June 2023, the enhanced and tested Birth App was launched and made available for download at no additional cost on Google Play and the App Store.

Previous studies have reported that mobile health (mHealth) apps designed to prepare women for the forthcoming labor and birth can be beneficial in terms of a better experience and labor outcome [16-18]. Fleming and colleagues [19] demonstrated the importance of ensuring credible electronic links, cell phone technology, videos, and access to hospital websites, which are created by the health care provider to educate expectant parents. Evans and colleagues [20] also highlighted the need for mobile apps developed in cocreation with users, researchers, and developers. They also emphasize that in maternity care, providers face significant challenges in identifying and endorsing digital health apps due to a lack of transparency regarding their evidence base, development methodology, and clinical validation. However, only a few evidence-based and codesigned apps have been developed and are available on the market [21].

The advent of digital health technologies offering unprecedented opportunities for patient engagement and monitoring is rising, and mHealth apps have gained attention in various fields to improve women’s health [22]. However, the proliferation of such apps has not always been accompanied by rigorous scientific validation, raising concerns regarding their efficacy and safety [23]. A review of mobile apps for women with anxiety in pregnancy showed that a majority of the apps (n=39) offered exclusively mind-body techniques and only a few offered informational support. A few of the apps (21%) reported involvement from professionals during development, and only one presented empirical evidence supporting effectiveness and user acceptability [20].

Apps can offer access to digital education and training programs, enabling women and their partners to gain new skills regardless of their geographical location, which can be particularly beneficial for women living in rural or remote areas. More knowledge is needed about how evidence-informed apps can be a complement to usual customary antenatal education [21].

Therefore, the aim of the project was to investigate whether women using the Birth App during pregnancy and childbirth experienced less distress during early labor, compared to women who received standard antenatal care. We hypothesized that women assigned to the Birth App groups would experience lower emotional stress during early labor compared to a control group that did not have access to the app. In addition, we hypothesized that women assigned to the Birth App Plus group would have additional benefits from using the app.

## Methods

### Swedish Study Setting

Midwives in Sweden have an autonomous role as primary care providers for women with uncomplicated pregnancies, labor, and birth, while obstetricians are consulted and responsible if complications arise. The antenatal care operates through community-based public health clinics, with midwives serving as the primary caregivers. Health education is a crucial aspect of prenatal care, focusing on lifestyle changes during pregnancy, and parental education is offered mainly to first-time parents. Almost all Swedish women give birth in hospitals, and maternity care is publicly funded [24].

### Trial Design

This RCT applied online recruiting in a nonblinded 3-part blended care model with a 1:1:1 randomization design. Group 1: Women assigned to the Birth App intervention. Group 2: Women assigned to Birth App Plus, combining the app with in-person additional midwifery contacts. Group 3: The control group received standard antenatal care, based on the preparations available at the antenatal care clinic where the woman was enrolled for pregnancy checkups.

### Recruitment

Pregnant nulliparous women were informed and invited to participate in the research study through a national invitation on the social media platforms Facebook and Instagram via paid advertising. Eligibility criteria included: nulliparous women planning to undergo a vaginal birth, pregnancy between 25+0 and 35+6 weeks at the time of registration, ability to speak, read, and understand Swedish, and access to a smartphone or tablet. Women interested in participating reported their interest on the research website. After receiving information about regulations according to the General Data Protection Regulation (GDPR) and signing digital informed consent, they were automatically linked to the baseline digital questionnaire in the Research Electronic Data Capture system (REDCap; Vanderbilt University) [25]. Thereafter, women were randomized by one of the research midwives using a block randomization of 6 with a computer-generated allocation list in Excel. Next, participating women were informed about their allocated group by email and SMS from the research midwives.

### Calculation of Sample Size

The aim of this study was to investigate whether women using the Birth App during pregnancy and childbirth experienced less distress during early labor, compared to women not using the app. The level of emotional distress has been tested in previous literature using ELEQ in different settings [26,27]. The primary outcome measure of this study was the Emotional Distress domain in the Swedish Early Labor Experience (SWE-ELEQ-PP). The calculation of sample size in the power analysis was guided by a study by Ängeby et al [4] which demonstrated significant differences in emotional distress among first-time mothers who had to leave the labor ward in early labor. Women who were dissatisfied about leaving the labor ward in early labor (n=43) scored significantly higher regarding emotional distress compared to the women who were content (n=54), mean 3.00 (SD 1.16) versus mean 2.58 (SD 1.01; *P*=.04) [4].

The sample size was calculated using IBM SPSS version 28. The hypothesis was that the Birth App could enhance women’s sense of security, thereby reducing stress and anxiety during early labor. The difference of 0.42 in the mean of emotional distress scores was used to guide the effect size for the power analysis. With a power level of 80% and a significance level of *P*<.05, the required sample size was calculated to be 82 participants in each group to detect a significant difference in the primary outcome between one of the intervention groups and the control group. The significant difference is calculated between the control group and the 2 experimental groups. Previous research with the targeted group of pregnant women has shown a 50% dropout rate, necessitating the inclusion of 160 participants in each group for sufficient statistical analysis [28]. Therefore, the total number of participants needed for the study was 480 women. In addition, participants who underwent induction of labor and did not spend time at home during early labor were thus unable to contribute to the primary analysis. In Sweden, approximately 25% of nulliparous women undergo induction, which needs to be considered as well for power estimation [29].

### Interventions

#### Intervention Group Birth App

Participants randomized to the Birth App group received an email with personalized instructions for downloading the app via TestFlight for iPhone users and Google Play for Android users. Each participant was given a unique personal code from a pregenerated list, consisting of 4 capital letters and 4 numbers. No additional follow-up was provided thereafter. The app has 2 parts: one for education and practical exercises, and 1 for use during actual labor. It aims to boost women’s self-efficacy and sense of security. The partner’s involvement is emphasized, with a dedicated section for nonpharmacological pain relief methods. The app provides information on the partner’s supportive role and includes exercises on contraction signs, closeness, and various pressure techniques to help the laboring woman during childbirth [14].

#### Intervention Group Birth App Plus

Participants randomized to the Birth App Plus group received personalized instructions identical to those given to the Birth App group. In addition, they were contacted by a research midwife via SMS, email, or phone conversations 2 weeks after enrollment, based on their preferred contact method. During the initial contact, questions regarding the app’s use and usability were addressed. The midwife was also available to answer other questions related to the method or its use during pregnancy. Topics such as feelings toward the forthcoming birth, coping ability, and partner support were discussed. This conversation aimed to strengthen the effectiveness of app usage and thereby enhance the outcomes. A second follow-up contact was conducted 2‐6 weeks after the first contact, serving as a follow-up to the previous conversation.

#### Control Group

Participants in the control group received usual antenatal care, where antenatal education is integrated into the care and provided free of charge by midwives and offered to all primiparous women and their partners.

### Questionnaire at Baseline

Participants completed a web-based questionnaire in REDCap prior to randomization, with baseline data accordingly.

#### Demographic Characteristics

Participating women provided information about age, marital status, level of education, profession, country of birth, as well as information concerning their physical and mental health.

#### Psychological Traits Measures

Using various psychological assessment tools at enrollment allows us to control for personality traits, ensuring these traits do not confound the study results. This approach enhances the validity and reliability of the study findings by accounting for individual differences that might influence responses to the intervention.

Sense of Coherence-13 (SOC) [30] was used to examine the resources of promoting individual health, comprising (1) comprehensibility, (2) manageability, and (3) meaningfulness dimensions [31]. The scale can be used as a continuous variable from 13 to 91, or categorized into low (<60), moderate (61-75), or high (>76) SOC [32]. A strong sense of coherence helps individuals mobilize resources to cope with stressors effectively, contributing to better health outcomes and a higher quality of life.

The Swedish Childbirth Self-Efficacy Inventory (Swe-CBSEI) [33] is a prevalidated, self-report instrument that measures an individual’s expectancies of coping with childbirth and describes an individual’s belief in their own ability to behave in a particular way in a specific situation [27]. The scale measures 2 different subscales during active labor: outcome expectancy (O-AL) and self-efficacy expectancy (E-AL), and a higher value represents a higher degree of E-AL, ranging from 15 to 150. It measures a person’s belief in their capacity to act effectively in specific childbirth-related situations.

The Fear of Birth Scale (FOBS) [34] was used to measure fear of birth. The FOBS is based on 2 visual analog scales from 1 to 100 for identifying fear of birth during pregnancy, and a cutoff value of >60 is normally used for identifying women with fear of birth [34]. High scores on FOBS indicate significant fear of childbirth, which can lead to increased anxiety, stress, and potential negative birth experiences. We used FOBS to measure fear of childbirth at baseline and follow-up, with questions adapted to assess fear retrospectively and for future births. This approach allowed us to track changes in fear over time and create a composite variable to categorize fear levels across different time points.

### Questionnaire at Follow-Up With Outcome Measures

One month postpartum, participants received a link to a follow-up questionnaire in REDCap.

The primary outcome was emotional distress in early labor. To address the primary outcome, SWE-ELEQ-PP [4] was used. The questionnaire was designed to measure women’s experience during early labor. The questionnaire covers 3 subscales: emotional distress (6 items), emotional well-being (7 items), and experiences of midwifery care (10 items). Emotional well-being and experiences of midwifery care are ranked from 1 to 5, meaning a higher value represents a more positive value. The subscale Emotional distress, ranked from 15, meaning a higher value represents a higher distress, was used as the primary outcome.

The questionnaire included study-specific questions about childbirth events such as labor onset, hours in labor before hospital admission, pain relief methods used during labor, and birthing mode. In addition, questions about the gestational week at birth, the baby’s care in the Neonatal Intensive Care Unit, and the maternity clinic where the birth took place.

The secondary outcomes were mode of birth, emotional well-being in early labor, and midwifery support during early labor (SWE-ELEQ-PP), childbirth experience, support from partner, pain relief methods, and fear of birth in a potential future birth.

The Childbirth Experience Questionnaire (CEQ) [35] was used to measure the total multidimensional childbirth experience. CEQ is developed and validated in Sweden and represents 4 domains or subscales of childbirth experience. Own capacity (8 items), perceived safety (6 items), professional support (5 items), and participation (3 items). Higher values represent a more positive experience in all subscales.

The Birth Companion Support Questionnaire (BCSQ) was used to measure women’s perceptions of companion support during childbirth, with 2 subscales: emotional support (8 items) and tangible support (6 items). Ranging from 1 to 4, with a higher value representing a higher perceived support from the partner [36].

The FOBS, rephrased as “when thinking about potential future birth,” was used to measure fear in forthcoming births. The scale ranges from 1 to 100, and a higher value represents a higher degree of fear in forthcoming births [37].

### Statistical Methods

Descriptive statistics for each group, such as mean, SD, median, and frequencies for categorical variables, were initially compiled. The chi-square test was used to examine differences between groups for categorical variables by comparing observed frequencies with expected frequencies to determine whether there was a statistically significant difference. ANOVA was used to compare means between the 3 groups for continuous variables. If ANOVA showed a significant difference, the Tukey post hoc test was used to identify group differences. Sensitivity testing was conducted by analyzing subgroups or using alternative statistical methods to explore differences between the intervention groups and the control group.

Pairwise testing was used to explore differences in the FOBS scores before and after childbirth. This statistical method allows for the comparison of each participant’s scores at 2 different time points, thereby accounting for individual variability and providing a more accurate assessment of changes over time. All outcomes were analyzed according to the intention-to-treat principle. Since the primary outcome was emotional distress during early labor, only women with spontaneous labor onset were included in the analysis of primary and secondary outcomes. Women with induced labor were excluded, since they were hospitalized before labor onset. The data were analyzed using IBM SPSS Statistics version 28, with a *P*<.05 considered statistically significant [38].

### Ethical Considerations

This study was approved by the Swedish Ethical Board (2021‐03028) and registered in ClinicalTrials.gov (NCT05122390), and the first version of the protocol was uploaded on November 16, 2021. No deviations from the registered protocol occurred. All participating women provided informed consent via REDCap after receiving study information and information about GDPR. To ensure confidentiality of the participants, all registered names and emails were replaced by a system-generated number provided in REDCap. This number was used to integrate their individual codes in the app, and data from the app was transferred to the researcher via a secure portal at Karlstad University, Sunet Drive. Participants completing the follow-up questionnaire received a gift card valued at SEK 200 (US $20.96) for use in a supermarket. No potential risks to participants were identified in this trial.

## Results

**Figure 1.**
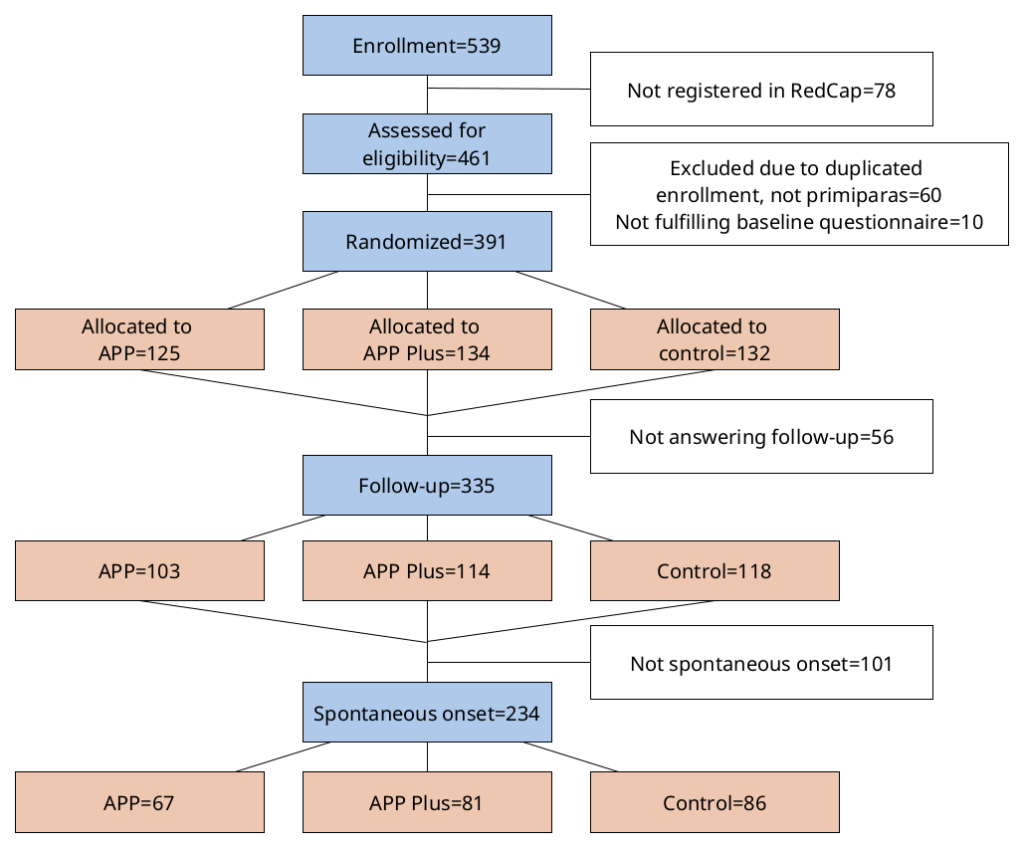
Flowchart of participants. REDCap: Research Electronic Data Capture.

Digital advertisements via social media were conducted from October 15, 2022, to March 15, 2023. A total of 539 women registered for participation in the study website. Following redirection to REDCap, 461 women were assessed for eligibility and 391 women were allocated to the study (Figure 1).

A total of 391 women completed the baseline questionnaire and 335 women responded to the questionnaire 1 month postpartum, yielding a response rate of 85.6%. At follow-up, 103 women in the Birth App group, 114 women in the Birth App Plus group, and 118 women in the control group completed the questionnaire. The mean age of the respondents was 31.30 (SD 3.70), 30.89 (SD 3.75), and 30.89 (SD 3.81) years, respectively. Most participants lived with a partner, had a university education, and were born in Sweden. Participants in all 3 groups reported similar values for the SOC, the CBSEI, and the FOBS at baseline. More than half of the participants attended antenatal birth classes during pregnancy. No statistically significant differences were observed between the intervention and control groups at baseline. No side effects such as unplanned out-of-hospital births were identified during the trial (Table 1).

Participating women gave birth in all regions of Sweden. Most women gave birth in the Stockholm-Gotland region (100/335, 29.8%), followed by the Mid-Sweden region (86/335, 25.6%), south-east region (55/335, 16.4%), western region (34/335, 10.2%) and least in northern region (28/335, 8.4%) and southern region (27/335, 8.1%).

**Table 1.** Characteristics of the participants (N=391).

	Birth App (n=125)	Birth App Plus (n=134)	Control (n=132)	*P* value
Age, mean (SD)	31.30 (3.70)	30.89 (3.75)	30.89 (3.81)	.87^a^
Highest education level, n (%)				.19^a^
Primary or high school	13 (10.6)	23 (17.2)	14 (10.9)	
College or University	110 (89.4)	111 (82.8)	115 (89.1)	
Marital status, n (%)				.68^a^
Living with partner	120 (97.6)	132 (98.5)	125 (96.9)	
Living alone	3 (.4)	2 (1.5)	4 (3.1)	
Country of birth, n (%)				.14^a^
Sweden	103 (83.7)	223 (91.8)	113 (87.6)	
Outside of Sweden	20 (16.3)	11 (8.2)	16 (12.4)	
SOC^b^, mean (SD)	64.9 (7.65)	64.5 (9.24)	64.0 (9.42)	.68^c^
CBSEI^d^, mean (SD)				
O-AL^e^	97.50 (22.89)	95.58 (25.21)	95.64 (23.06)	.77^c^
E-AL^f^	85.07 (20.85)	84.47 (26.06)	86.52 (26.27)	.78^c^
FOBS^g^, mean (SD)	46.22 (24.42)	46.05 (24.41)	48.48 (23.31)	.67^c^
FOBS >60, n (%)	37 (31.4)	44 (33.1)	41 (33.1)	.94^a^
Birth preparation course (answered after birth), n (%)				
Yes	71 (56.8)	87 (64.9)	83 (62.9)	.35^a^

a Pearson *χ*2 test.

b SOC: Sense of Coherence.

c ANOVA, post hoc Tukey.

d CBSEI: Childbirth Self-Efficacy Inventory.

e O-AL: outcome expectancy.

f E-AL: self-efficacy expectancy.

g FOBS: Fear of Birth Scale.

Most participants experienced a spontaneous onset of labor across all groups, with no significant statistical differences. A small number of women (n=8) had an elective cesarean section and were therefore removed from the analysis. Approximately 25% of participants had induced labor, with no significant differences between groups.

Women in the intervention groups remained at home longer during early labor compared to women in the control group, although this difference was not statistically significant (*P*=.32) (Table 2). To assess the robustness, we conducted a sensitivity analysis by combining the 2 intervention groups. The result showed a mean difference of 2.71 (CI −1.51 to 6.93; *P=*.20). The mode of birth showed no significant differences between the groups. Most women used pharmacological pain relief during labor (Entonox and Epidurals) with no significant differences between groups.

**Table 2.** Labor outcome at follow-up (n=335).

	App (n=103)	App Plus (n=114)	Control (n=118)	*P* value
Labor onset, n (%)				
Spontaneous	68 (54.4)	83 (61.9)	86 (65.2)	.15^a^
Induction	34 (27.2)	29 (21.6)	27 (20.5)	
Elective cesarean section	1 (0.8)	2 (1.5)	5 (3.8)	
Length of latent phase before admission in hours, mean (SD)	16.8 (20.45)	14.5 (16.82)	12.9 (15.99)	.31^a^
Birth mode, n (%)				
Spontaneous vaginal birth	71 (69.6)	81 (71.1)	93 (78.8)	.43^b^
Vacuum extraction	14 (13.7)	13 (11.4)	8 (6.8)	
Acute cesarean section	17 (16.7)	20 (17.5)	17 (14.4)	
Pharmacological pain relief, n (%)				
Yes	88 (70.4)	98 (73.1)	100 (75.8)	.47^b^

a ANOVA, post hoc test Tukey.

b Pearson *χ*2 test.

For the primary outcome, emotional distress in early labor showed similar mean values across groups (Birth App: mean 2.42, SD 0.78; Birth App Plus: mean 2.29, SD 0.84; and control group: mean 2.45, SD 0.83), with a *P* value of .44 (Table 3). A sensitivity analysis, comparing intervention groups to the control group, showed the mean difference of −0.085 (CI −0.276 to 0.106; *P=*.38). For secondary outcomes, women in all groups reported nearly identical values for emotional well-being and midwifery support, as measured by the 2 subscales in the SWE-ELEQ-PP. Regarding the dimensions in the CEQ, including own capacity, professional support, and participation, similar mean values were reported across the groups. However, for the subscale perceived safety, women in the APP Plus group scored higher (mean 3.28, SD 0.62), compared to the Birth App (mean 3.14, SD 0.62) and the control group (mean 3.14, SD 0.71), though these differences were not statistically significant. When measuring partner support, no statistical differences were found between groups. All women rated their emotional support statistically significantly higher than tangible support, with similar mean values across all groups.

**Table 3.** Childbirth experience for women with spontaneous onset of labor (n=234).

	Birth App (n=67)	Birth Plus (n=81)	Control (n=86)	*P* value
SWE-ELEQ^a^, mean (SD)				
Emotional distress	2.42 (0.78)	2.29 (0.84)	2.45 (0.83)	.45^b^
Emotional well-being	3.79 (0.68)	3.91 (0.70)	3.83 (0.73)	.54^b^
Midwifery care	4.39 (0.66)	4.32 (0.69)	4.38 (0.82)	.91^b^
CEQ^c^, mean (SD)				
Own capacity	2.66 (0.54)	2.79 (0.56)	2.76 (0.54)	.39^b^
Professional support	3.62 (0.59)	3.61 (0.47)	3.65 (0.53)	.90^b^
Perceived safety	3.14 (0.62)	3.28 (0.62)	3.14 (0.71)	.41^b^
Participation	3.25 (0.62)	3.39 (0.64)	3.33 (0.67)	.47^b^
SWE-BCSQ^d^, mean (SD)				
Emotional support	3.81 (0.27)	3.80 (0.33)	3.74 (0.45)	.33^b^
Tangible support	3.56 (0.60)	3.53 (0.59)	3.53 (0.64)	.63^b^

a SWE-ELEQ: Swedish Early Labor Experience Questionnaire.

b ANOVA, post hoc Tukey. Values are presented if a statistically significant difference occurs.

c CEQ: Childbirth Experience Questionnaire.

d SWE-BCSQ: Sweden Birth Companion Support Questionnaire.

When assessing fear at baseline, women in all groups scored similar mean values, Birth App (mean 45.7, SD 24.6), Birth App Plus (mean 44.9, SD 25.3), and control group (mean 47.0, SD 22.6; *P*=.86; Table 4). When assessing their fear of childbirth in forthcoming births, the intervention groups indicated lower mean values than the control group (mean 32.78, SD 28.43 and mean 31.17, SD 31.40 vs mean 38.47, SD 32.17; *P*=.07). In a sensitivity test, using pairwise testing, we were able to control for individual differences and obtain a clearer understanding of the impact of the intervention on participants’ fear of childbirth. It revealed a statistically significant difference for both intervention groups (*P=*.002 and *P*<.001) with a medium effect size according to Cohen *d* (0.40 and 0.47), while the control group showed a nonsignificant value (*P=*.08).

**Table 4.** Fear of birth repeated measures for women with spontaneous onset of labor (n=234).

Measure	Birth App (n=67)	Birth App Plus (n=81)	Control (n=86)	*P*^a^ value
FOBS^b^, mean (SD)				
Baseline	45.65 (24.56)	44.92 (25.27)	46.96 (22.64)	.86
Forthcoming birth	32.78 (28.43)	31.17 (31.40)	38.47 (32.17)	.06
Baseline vs forthcoming birth				
Mean difference (95% CI)	13.53 (5.12 to 21.92)	14.59 (7.75 to 21.42)	6.78 (–0.95 to 14.53)	—^c^
*t* value (*df*)	3.21 (62)	4.24 (81)	1.74 (75)	—
*P* value^d^	.002	<.001	.08	—
Cohen *d*	0.40	0.47	0.20	—

a ANOVA, post hoc Tukey

b FOBS: Fear of Birth Scale.

c Not applicable.

d Paired sample effect sizes*.*

## Discussion

### Principal Findings and Comparison With Prior Work

Our study compared women using the Birth App during pregnancy and childbirth with customary antenatal care. Women in the Birth App Plus group, which included additional midwifery support, reported less emotional distress during early labor, but not at a statistically significant level. No side effects or potential risk factors were identified in the RCT.

Our hypothesis that women in the intervention groups experienced less distress in early labor could not be established. Previous research from other studies testing different types of interventions aimed at reducing early labor distress did not show statistically significant differences either [27,39,40]. Women in the intervention groups stayed at home slightly longer during early labor compared to women in the control group, although with considerable variation. This suggests that the app functions can be a useful tool for pregnant women and increase coping and management ability during early labor. Similar results were also identified in our previous pilot study, showing high usability and usefulness [14].

For secondary outcomes, the 4 different dimensions of childbirth were similar in all groups. However, for the subscale perceived safety, women in the APP Plus group scored higher, compared to the other groups, although not reaching a statistically significant difference. Dencker and colleagues [35] demonstrated that nonvaginal births, oxytocin augmentation, and longer labor negatively affected all subscales. For the subscale perceived safety, an intercorrelation between fear, sense of security, and negative memories from childbirth was established [35]. A systematic review indicated that mindfulness-based interventions could reduce fear of childbirth and promote self-efficacy [41]. Another study by Carlsson et al [42] found that self-efficacy correlated with reduced use of epidural analgesia among primiparous women, which may reflect their ability to exert control and experience safety as observed in this study.

In this study, fear of forthcoming births was significantly lower in both intervention groups compared to the control group. By using pairwise testing, we were able to control individual differences and obtain a clearer understanding of the impact of the intervention on participants’ fear of childbirth. Klabbers et al [43] showed in an RCT that haptotherapy could significantly reduce fear of birth compared to psychoeducation via the internet and usual care. Haptotherapy is designed to promote a more positive attitude in pregnant women and change cognitive appraisal to improve readiness for childbirth [44]. The Birth App, tested in this study, is based on the Birth Without Fear method, which also focuses on cognitive aspects, aiming to strengthen both physical and emotional capacity [15]. Other studies have shown the importance of tested and valid apps for pregnant women, especially for women with anxiety during pregnancy [20] and emphasize mHealth apps to align with pregnant women’s preferences for an mHealth app during pregnancy [45,46].

A strength of this study is that the 3 groups were equal in background characteristics. The similarity in educational level across all groups increases the trustworthiness of our results. Another strength is the high response rate, with 85% of participating women completing the postpartum questionnaire, consistent across all groups. The majority had a spontaneous onset of labor, and the group with induced labor (25%) was identical to a national sample [29]. In addition, online recruitment allowed women from all parts of Sweden to participate, providing a broad sample of primiparous women. Participating women gave birth in almost all maternity clinics in Sweden, which is a strength regarding generalization. The rate of acute cesarean section was similar in all groups, and the 16.2% rate of cesarean sections is lower than the average in Sweden [29], and the reason for this can only be speculated about. The participating women were highly educated, slightly older, and interested in using different antenatal preparation methods, which possibly can explain the result.

### Limitations

This study also has some limitations. The participating women had a higher level of education compared to the Swedish female population of the same age. Sampling through social networks and the high educational level of participants may limit applicability to vulnerable populations since previous research has shown that women with lower education levels are less likely to use digital information [47].

Another limitation is that most women in all groups participated in various antenatal classes, which may have provided additional education that we could not control for, potentially affecting the results. The control group could have accessed other digital tools or private antenatal classes, which could have diluted the effects. The participants were not restricted or prohibited from using other accessible apps, and it is possible that women randomized to the control group were dissatisfied with their enrollment and, therefore, used another app or attended private antenatal classes instead. Future studies should consider strategies to monitor or control app usage tracking to better isolate the effects of the intervention under investigation.

Another aspect is the number of women completing the research: Birth App group, n=103; Birth App Plus group, n=114; and control group, n=118. In the power calculation, we accounted for 160 participants in each group. However, the attrition rate was lower than expected, and the induction rate was accounted for; therefore, the expected power in all groups was achieved.

We also need to consider the fact that women may have used the app in terms of time differently, which may affect the result. However, in this study, the time-usage variable was not addressed in the planned RCT protocol uploaded in ClinicalTrials.gov (ref. no. NCT05122390). Future studies should incorporate mechanisms to track and analyze app usage patterns to better understand how engagement correlates with outcomes.

### Conclusions

The trial evaluating the Birth App yielded important findings. The hypothesis that the Birth App would reduce emotional distress in early labor was not confirmed in this study. Our results indicate that the app, in conjunction with additional midwifery support, can serve as a valuable tool for pregnant women and their partners, enhancing their confidence during early labor, especially for primiparous women with a fear of birth, which warrants further investigation.

## Supplementary material

10.2196/72807 Checklist 1 CONSORT-eHealth checklist (V 1.6.2).

## Abbreviations

BCSQ: Birth Companion Support Questionnaire
CEQ: Childbirth Experience Questionnaire
E-AL: self-efficacy expectancy
ELEQ: Early Labor Experience Questionnaire
FOBS: Fear of Birth Scale
GDPR: General Data Protection Regulation
O-AL: outcome expectancy
RCT: randomized controlled trial
REDCap: Research Electronic Data Capture
SOC: Sense of Coherence
Swe-CBSEI: Swedish Childbirth Self-Efficacy Inventory
