# Correction: Testing the ELSA Birth App During Pregnancy and Labor for Primiparous Women: Randomized Controlled Trial

**DOI:** 10.2196/86193

**Published:** 2025-10-24

**Authors:** Karin Ängeby, Margareta Johansson, Elin Børøsund, Cecilie Varsi, Leonardo Horn Iwaya, Anna Nordin

**Affiliations:** 1 Department of Health Science, Faculty of Health, Science, and Technology, Karlstad University, Universitetsgatan 2, Karlstad, 65188, Sweden, 46 703579767; 2 Women's Department and Centre for Clinical Research and Education, Region Värmland, Karlstad, Sweden; 3 Department of Women’s and Children’s Health, Uppsala University, Uppsala, Sweden; 4 Department of Women’s Health Care, Akademiska University Hospital, Uppsala, Sweden; 5 Department of Digital Health Research, Division of Medicine, Oslo University Hospital, Oslo, Norway; 6 Department of Nursing and Health Sciences, Faculty of Health and Social Sciences, University of South-Eastern Norway, Drammen, Norway; 7 Faculty of Health and Social Sciences, University of South-Eastern Norway, Drammen, Norway; 8 Department of Mathematics and Computer Science, Faculty of Health, Science, and Technology, Karlstad University, Karlstad, Sweden

In “Testing the ELSA Birth App During Pregnancy and Labor for Primiparous Women: Randomized Controlled Trial” [1], the authors noted one error in the affiliations.

Author AN was previously listed with no affiliation. Her affiliation has been revised to:

^1^Department of Health Science, Faculty of Health, Science, and Technology, Karlstad University, Karlstad, Sweden

The correction will appear in the online version of the paper on the JMIR Publications website, together with the publication of this correction notice. Because this was made after submission to PubMed, PubMed Central, and other full-text repositories, the corrected article has also been resubmitted to those repositories.
