# Supplementary material for: Testing the ELSA Birth App During Pregnancy and Labor for Primiparous Women: Randomized Controlled Trial
Source: J Med Internet Res. 2025 Oct 16;27:e72807. doi: 10.2196/72807 (PMC12530693; doi:10.2196/72807)
Supplement: Checklist 1 [file jmir-v27-e72807-s001.pdf]

CONSORT-EHEALTH Checklist V1.6.2 Report	Manuscript Number	72807
**(based on CONSORT-EHEALTH V1.6), available at [http://tinyurl.com/consort-ehealth-v1-6].**		
**Date completed**		
9/8/2025 6:54:11		
**by**		
Karin Ängeby		
TITLE		
**1a-i) Identify the mode of delivery in the title**		
**1a-ii) Non-web-based components or important co-interventions in title**		
Testing "Early Labour Support APP" during pregnancy and labour for primiparous women: a "Randomized Controlled Trial"		
**1a-iii) Primary condition or target group in the title**		
ABSTRACT		
**1b-i) Key features/functionalities/components of the intervention and comparator in the METHODS section of the ABSTRACT**		
**1b-ii) Level of human involvement in the METHODS section of the ABSTRACT**		
Like this: Early labor is often managed at home without professional support. The Birth APP is a mHealth application designed to support women during early labor.		
**1b-iii) Open vs. closed, web-based (self-assessment) vs. face-to-face assessments in the METHODS section of the ABSTRACT**		
Like this: This RCT used online recruiting in a non-blinded three-part blended care model with 1:1:1 randomization. Group 1: Birth APP intervention, "group 2: Birth APP Plus, combining the app with in-person additional midwifery contacts", and group 3: Control group receiving standard antenatal care.		
**1b-iv) RESULTS section in abstract must contain use data**		
Like this: Pregnant nulliparous women were invited via social media. Eligibility criteria were: nulliparity, planning a vaginal birth, from gestational week 25+0 to 35+6 weeks, proficiency in understanding Swedish, and having access to a smartphone or tablet.<br><br>Like this:A total of 391 women completed the baseline questionnaire and were included in the study (group 1, n=118; group 2, n=114; group 3, n=118). Of these, 334 women responded to the questionnaire 1 month postpartum, yielding a response rate of 85.4%		
**1b-v) CONCLUSIONS/DISCUSSION in abstract for negative trials**		
Like this: A total of 391 women completed the baseline questionnaire, and 335 women responded to the questionnaire 1 month postpartum, yielding a response rate of 85.4%. At follow-up, 103 women in the Birth APP group, 114 women in the Birth APP Plus group, and 118 women in the control group completed the questionnaire.		
INTRODUCTION		
**2a-i) Problem and the type of system/solution**		
**2a-ii) Scientific background, rationale: What is known about the (type of) system**		
Like this: To enhance women's confidence to remain at home during early labor, adequate preparation for managing pain in early labor is important [1], as feeling anxious increases the perception of pain [2].<br><br>The Birth Without Fear© method was later conceptualized in a digital application (app), and a first version was tested in a feasibility pilot study. The result showed that the app was perceived as useful and appreciated by women and suitable for efficacy testing in an RCT [14].		
METHODS		
**3a) CONSORT: Description of trial design (such as parallel, factorial) including allocation ratio**		
**3b) CONSORT: Important changes to methods after trial commencement (such as eligibility criteria), with reasons**		
Like this: Therefore, the aim of the project was to investigate whether women using the Birth APP during pregnancy and childbirth experienced less distress during early labor, compared to women who received standard antenatal care. We hypothesized that women assigned to the Birth APP groups would experience lower emotional stress during early labor compared to a control group that did not have access to the app. Additionally, we hypothesized that women assigned to the Birth APP Plus group would have additional benefits from using the app.		
**3b-i) Bug fixes, Downtimes, Content Changes**		
**4a) CONSORT: Eligibility criteria for participants**		
Like this: This RCT applied online recruiting in a non-blinded 3-part blended care model with a 1:1:1 randomization design. Group 1: Women assigned to the Birth APP intervention. Group 2: Women assigned to Birth APP Plus, combining the app with in-person additional midwifery contacts. Group 3: The control group received standard antenatal care, based on the preparations available at the antenatal care clinic where the woman was enrolled for pregnancy checkups.		
**4a-i) Computer / Internet literacy**		
**4a-ii) Open vs. closed, web-based vs. face-to-face assessments:**		
Pregnant women are a group with high digital literacy.		
**4a-iii) Information giving during recruitment**		
Like this: Women interested in participating reported their interest on the research website.		
**4b) CONSORT: Settings and locations where the data were collected**		
No changes with eligibility criteria were done after trial commencement.		
**4b-i) Report if outcomes were (self-)assessed through online questionnaires**		
**4b-ii) Report how institutional affiliations are displayed**		

Like this:Participants completed a web-based questionnaire in RedCap prior to randomization, with baseline data accordingly.<br><br>Using various psychological assessment tools at enrolment allows us to control for personality traits, ensuring these traits do not confound the study results. This approach enhances the validity and reliability of the study findings by accounting for individual differences that might influence responses to the intervention.<br>Sense of Coherence-13 (SOC) [30], was used to examine the resource of promoting individual health, composing (a) comprehensibility, (b) manageability, and (c) meaningfulness dimensions [31]. The scale can be used as a continuous variable from 13-91, or categorized into low (<60), moderate (61-75), or high (>76) SOC [32]. A strong sense of coherence helps individuals mobilize resources to cope with stressors effectively, contributing to better health outcomes and a higher quality of life.<br>The Swedish Childbirth Self-Efficacy Inventory (Swe-CBSEI) [33], is a pre-validated, self-report instrument that measures an individual’s expectancies of coping with childbirth and describes an individual’s belief in their own ability to behave in a particular way in a specific situation [27]. The scale measures 2 different subscales during active labor: Outcome expectancy (O-AL) and Self-Efficacy expectancy (E-AL), and a higher value represents a higher degree of self-efficacy expectancy, ranging from 15-150. It measures a person's belief in their capacity to act effectively in specific childbirth-related situations.<br>The Fear of Birth-scale (FOBS) [34] was used to measure fear of birth. The FOBS scale is based on 2 visual analogue scales from 1-100 for identifying fear of birth during pregnancy, and a cut-off value of >60 is normally used for identifying women with fear of birth [34]. High scores on FOBS indicate significant fear of childbirth, which can lead to increased anxiety, stress, and potential negative birth experiences. We used FOBS to measure fear of childbirth at baseline and follow-up, with questions adapted to assess fear retrospectively and for future births. This approach allowed us to track changes in fear over time and create a composite variable to categorize fear levels across different time points.		
**5) CONSORT: Describe the interventions for each group with sufficient details to allow replication, including how and when they were actually administered**		
**5-i) Mention names, credential, affiliations of the developers, sponsors, and owners**		
**5-ii) Describe the history/development process**		
**5-iii) Revisions and updating**		
Like this:The Birth Without Fear© method was later conceptualized in a digital application (app), and a first version was tested in a feasibility pilot study. The result showed that the app was perceived as useful and appreciated by women and suitable for efficacy testing in an RCT [14]. The company Birth by Heart led the app development, involving senior software developers with expertise in health and fitness apps, and an expert group to define the requirements. Regular stakeholder meetings ensured alignment, reviewed app versions, and incorporated feedback throughout the process. The app development process is more thoroughly described in another publication [15]. In June 2023, the enhanced and tested Birth APP was launched and made available for download at no additional cost on Google Play and the App Store.		
**5-iv) Quality assurance methods**		
**5-v) Ensure replicability by publishing the source code, and/or providing screenshots/screen-capture video, and/or providing flowcharts of the algorithms used**		
**5-vi) Digital preservation**		
**5-vii) Access**		
The app is available on the market now.		
**5-viii) Mode of delivery, features/functionalities/components of the intervention and comparator, and the theoretical framework**		
Like this: Participants randomized to the Birth APP group received an email with personalized instructions for downloading the app via TestFlight for iPhone users and Google Play for Android users. Each participant was given a unique personal code from a pre-generated list, consisting of 4 capital letters and 4 numbers.		
**5-ix) Describe use parameters**		
Like this:Participants randomized to the Birth APP Plus group received personalized instructions identical to those given to the Birth APP group. Additionally, they were contacted by a research midwife via SMS, email, or phone conversations 2 weeks after enrolment, based on their preferred contact method. During the initial contact, questions regarding the app's use and usability were addressed. The midwife was also available to answer other questions related to the method or its use during pregnancy. Topics such as feelings toward the forthcoming birth, coping ability, and partner support were discussed. This conversation aimed to strengthen the effectiveness of app usage and thereby enhance the outcomes. A second follow-up contact was conducted 2–6 weeks after the first contact, serving as a follow-up to the previous conversation.		
**5-x) Clarify the level of human involvement**		
**5-xi) Report any prompts/reminders used**		
Like this: Participants randomized to the Birth APP Plus group received personalized instructions identical to those given to the Birth APP group. Additionally, they were contacted by a research midwife via SMS, email, or phone conversations 2 weeks after enrolment, based on their preferred contact method. During the initial contact, questions regarding the app's use and usability were addressed. The midwife was also available to answer other questions related to the method or its use during pregnancy. Topics such as feelings toward the forthcoming birth, coping ability, and partner support were discussed. This conversation aimed to strengthen the effectiveness of app usage and thereby enhance the outcomes. A second follow-up contact was conducted 2–6 weeks after the first contact, serving as a follow-up to the previous conversation.		
**5-xii) Describe any co-interventions (incl. training/support)**		
The app was not giving any reminders.		
**6a) CONSORT: Completely defined pre-specified primary and secondary outcome measures, including how and when they were assessed**		
Like this: Eligibility criteria included: nulliparous women planning to undergo a vaginal birth, pregnancy between 25+0 and 35+6 weeks at the time of registration, ability to speak, read, and understand Swedish, and access to a smartphone or tablet.		
**6a-i) Online questionnaires: describe if they were validated for online use and apply CHERRIES items to describe how the questionnaires were designed/deployed**		
**6a-ii) Describe whether and how “use” (including intensity of use/dosage) was defined/measured/monitored**		
Only validated questionnaires were used and described above.		
**6a-iii) Describe whether, how, and when qualitative feedback from participants was obtained**		
The participants were only encouraged to use the app but not forced.		
**6b) CONSORT: Any changes to trial outcomes after the trial commenced, with reasons**		
Like this: Pregnant nulliparous women were informed and invited to participate in the research study through a national invitation on the social media platforms Facebook and Instagram via paid advertising. Eligibility criteria included: nulliparous women planning to undergo a vaginal birth, pregnancy between 25+0 and 35+6 weeks at the time of registration, ability to speak, read, and understand Swedish, and access to a smartphone or tablet.		
**7a) CONSORT: How sample size was determined**		
**7a-i) Describe whether and how expected attrition was taken into account when calculating the sample size**		
**7b) CONSORT: When applicable, explanation of any interim analyses and stopping guidelines**		

Like this:One month postpartum, participants received a link to a follow-up questionnaire in RedCap.<br>The primary outcome was emotional distress in early labor. To address the primary outcome, SWE-ELEQ-PP [4] was used. The questionnaire was designed to measure women's experience during early labor. The questionnaire covers 3 subscales: emotional distress (6 items), emotional well-being (7 items), and experiences of midwifery care (10 items). Emotional well-being and experiences of midwifery care are ranked from 1-5, meaning a higher value represents a more positive value. The subscale Emotional distress, ranked from 1-5, meaning a higher value represents a higher distress, was used as the primary outcome.<br>The questionnaire included study-specific questions about childbirth events such as labor onset, hours in labor before hospital admission, pain relief methods used during labor, and birthing mode. Additionally, questions about the gestational week at birth, the baby's care in the Neonatal Intensive Care Unit, and the maternity clinic where the birth took place.<br>The secondary outcomes were mode of birth, emotional well-being in early labor, and midwifery support during early labor (SWE-ELEQ-PP), childbirth experience, support from partner, pain relief methods, and fear of birth in a potential future birth.<br>The Childbirth Experience Questionnaire (CEQ) [35] was used to measure the total multidimensional childbirth experience. CEQ is developed and validated in Sweden and represents 4 domains or subscales of childbirth experience. Own capacity (8 items), perceived safety (6 items), professional support (5 items), and participation (3 items). Higher values represent a more positive experience in all subscales.<br>The Birth Companion Support Questionnaire (BCSQ) was used to measure women's perceptions of companion support during childbirth, with 2 subscales: emotional support (8 items) and tangible support (6 items). Ranging from 1-4, with a higher value representing a higher perceived support from the partner [36].<br>The FOBS scale, rephrased as "when thinking about potential future birth," was used to measure fear in forthcoming births. The scale ranges from 1-100, and a higher value represents a higher degree of fear in forthcoming births [37].		
**8a) CONSORT: Method used to generate the random allocation sequence**		
No changes were done due to high response rate.		
**8b) CONSORT: Type of randomisation; details of any restriction (such as blocking and block size)**		
Not relevant for this study since no side effects were identified.		
**9) CONSORT: Mechanism used to implement the random allocation sequence (such as sequentially numbered containers), describing any steps taken to conceal the sequence until interventions were assigned**		
Like this: This RCT applied online recruiting in a non-blinded 3-part blended care model with a 1:1:1 randomization design.		
**10) CONSORT: Who generated the random allocation sequence, who enrolled participants, and who assigned participants to interventions**		
Like this: Thereafter, women were randomized by one of the research midwives using a block randomization of 6 with a computer-generated allocation list in Excel. Next, participating women were informed about their allocated group by e-mail and short message service (SMS) from the research midwives.		
**11a) CONSORT: Blinding - If done, who was blinded after assignment to interventions (for example, participants, care providers, those assessing outcomes) and how**		
**11a-i) Specify who was blinded, and who wasn't**		
**11a-ii) Discuss e.g., whether participants knew which intervention was the "intervention of interest" and which one was the "comparator"**		
The intervention was not blinded. All participants were informed about their allocated group.		
**11b) CONSORT: If relevant, description of the similarity of interventions**		
Please read as stated above. One of the intervention groups had additionally midwifery support.		
**12a) CONSORT: Statistical methods used to compare groups for primary and secondary outcomes**		
The trial is registred in ClinicalTrials but no publication such as trial protocol is published.		
**12a-i) Imputation techniques to deal with attrition / missing values**		
**12b) CONSORT: Methods for additional analyses, such as subgroup analyses and adjusted analyses**		
The participants could use other sources of antenatal prepartation which is further discussed in the discussion section under limitations.		
RESULTS		
**13a) CONSORT: For each group, the numbers of participants who were randomly assigned, received intended treatment, and were analysed for the primary outcome**		
To clarify some issues i e ethical considerations.		
**13b) CONSORT: For each group, losses and exclusions after randomisation, together with reasons**		
Like this: Sensitivity testing was conducted by analyzing subgroups or using alternative statistical methods to explore differences between the intervention groups and the control group.<br><br>Pairwise testing was employed to explore differences in the FOBS scores before and after childbirth. This statistical method allows for the comparison of each participant's scores at 2 different time points, thereby accounting for individual variability and providing a more accurate assessment of changes over time. All outcomes were analyzed according to the intention-to-treat principle. Since the primary outcome was emotional distress during early labor, only women with spontaneous labor onset were included in the analysis of primary and secondary outcomes.		
**13b-i) Attrition diagram**		
**14a) CONSORT: Dates defining the periods of recruitment and follow-up**		
Like this: A total of 391 women completed the baseline questionnaire, and 334 women responded to the questionnaire 1 month postpartum, yielding a response rate of 85.4%. At follow-up, 103 women in the Birth APP group, 114 women in the Birth APP Plus group, and 118 women in the control group completed the questionnaire.<br><br>Participating women gave birth in all regions of Sweden. Most women gave birth in the Stockholm-Gotland region (100/334, 30.3%), followed by the Mid-Sweden region (86/334, 26.1%), south-east region (55/334, 16.7%), western region (34/334, 10.3%) and least in northern region (28/334, 8.5%) and southern region (27/334, 8.2%).<br>Most participants experienced a spontaneous onset of labor across all groups, with no significant statistical differences. A small number of women (n=8) had an elective caesarean section and were therefore removed from the analysis. Approximately 25% of participants had induced labor, with no significant differences between groups.		
**14a-i) Indicate if critical "secular events" fell into the study period**		
**14b) CONSORT: Why the trial ended or was stopped (early)**		
A flowchart over participants are included in the manuscript.		
**15) CONSORT: A table showing baseline demographic and clinical characteristics for each group**		
Like this: One month postpartum, participants received a link to a follow-up questionnaire in RedCap.		
**15-i) Report demographics associated with digital divide issues**		
**16a) CONSORT: For each group, number of participants (denominator) included in each analysis and whether the analysis was by original assigned groups**		
**16-i) Report multiple "denominators" and provide definitions**		
**16-ii) Primary analysis should be intent-to-treat**		
As stated above we did not include the amount of time spent in the app since this was not a psrt of the planned analysis.		
**17a) CONSORT: For each primary and secondary outcome, results for each group, and the estimated effect size and its precision (such as 95% confidence interval)**		
The trial was stopped when the calculated sample was fulfilled.		
**17a-i) Presentation of process outcomes such as metrics of use and intensity of use**		
**17b) CONSORT: For binary outcomes, presentation of both absolute and relative effect sizes is recommended**		
Please see table 1 and text in the manuscript.		

18) CONSORT: Results of any other analyses performed, including subgroup analyses and adjusted analyses, distinguishing pre-specified from exploratory

Like this:Women in the intervention groups remained at home longer during early labor compared to women in the control group, although this difference was not statistically significant (P=.316) (Table 2). To assess the robustness, we conducted a sensitivity analysis by combining the 2 intervention groups. The result showed a mean difference of 2.71, CI -1.51 to 6.93, P=.201. The mode of birth showed no significant differences between the groups. Most women used pharmacological pain relief during labor (Entonox, Epidurals) with no significant differences between groups.
For the primary outcome, emotional distress in early labor showed similar mean values across groups (Birth APP, M=2.42 [SD 0.78]; Birth APP Plus, M=2. 29 [SD 0.84]; and control group, M=2.45 [SD 0.83]). with a P-value of .435 (Table 3). A sensitivity analysis, comparing intervention groups to the control group, showed the mean difference -.085, CI -.276 to .106, P=.384. For secondary outcomes, women in all groups reported nearly identical values for emotional well-being and midwifery support, as measured by the two subscales in the SWE-ELEQ-PP. Regarding the dimensions in the CEQ, including own capacity, professional support, and participation, similar mean values were reported across the groups. However, for the subscale perceived safety, women in the APP Plus group scored higher (M=3.28 [SD 0.62]), compared to the Birth APP (M=3.14 [SD 0.62]) and the control group (M=3.14 [SD 0.71]), though these differences were not statistically significant. When measuring partner support, no statistical differences were found between groups. All women rated their emotional support statistically significantly higher than tangible support, with similar mean values across all groups.

18-i) Subgroup analysis of comparing only users

19) CONSORT: All important harms or unintended effects in each group

Like this: When assessing fear at baseline, women in all groups scored similar mean values, Birth APP (M=45.7 [SD 24.6]), Birth APP Plus (M=44.9 [SD 25.3]), and control group (M=47.0 [SD 22.6]) P=.862 (Table 4). When assessing their fear of childbirth in forthcoming births, the intervention groups indicated lower mean values than the control group (M=32.78/M=31.17 vs. M=38.47, P=.067). In a sensitivity test, using pairwise testing, we were able to control for individual differences and obtain a clearer understanding of the impact of the intervention on participants' fear of childbirth. It revealed a statistically significant difference for both intervention groups (P=.002 and P<.001) with a medium effect size according to Cohen d (0.40 and 0.47), while the control group showed a non-significant value (P=.085).

19-i) Include privacy breaches, technical problems

19-ii) Include qualitative feedback from participants or observations from staff/researchers

During the process no technical issues were identified.

DISCUSSION

20) CONSORT: Trial limitations, addressing sources of potential bias, imprecision, multiplicity of analyses

20-i) Typical limitations in ehealth trials

21) CONSORT: Generalisability (external validity, applicability) of the trial findings

21-i) Generalizability to other populations

21-ii) Discuss if there were elements in the RCT that would be different in a routine application setting

Like this: This study also has some limitations. The participating women had a higher level of education compared to the Swedish female population of the same age. Sampling through social networks and the high educational level of participants may limit applicability to vulnerable populations since previous research has shown that women with lower education levels are less likely to use digital information [48].

22) CONSORT: Interpretation consistent with results, balancing benefits and harms, and considering other relevant evidence

22-i) Restate study questions and summarize the answers suggested by the data, starting with primary outcomes and process outcomes (use)

22-ii) Highlight unanswered new questions, suggest future research

Like this:Our study compared women using the Birth APP during pregnancy and childbirth with customary antenatal care. Women in the Birth APP Plus group, which included additional midwifery support, reported less emotional distress during early labor, but not at a statistically significant level. No side effects or potential risk factors were identified in the RCT.
Our hypothesis that women in the intervention groups experienced less distress in early labor could not be established. Previous research from other studies testing different types of interventions aiming at reducing early labor distress, did not show statistically significant differences either [27, 39, 40]. Women in the intervention groups stayed at home slightly longer during early labor compared to women in the control group, although with considerable variation. This suggests that the app functions can be a useful tool for pregnant women and increase coping and management ability during early labor. Similar results were also identified in our previous pilot study, showing high usability and usefulness [14].
For secondary outcomes, the 4 different dimensions of childbirth were similar in all groups. However, for the subscale perceived safety, women in the APP Plus group scored higher, compared to the other groups, although not reaching a statistically significant difference. Dencker and colleagues [35] demonstrated that non-vaginal births, oxytocin augmentation, and longer labors negatively affected all subscales. For the subscale perceived safety, an intercorrelation between fear, sense of security, and negative memories from the childbirth was established [35]. A systematic review indicated that mindfulness-based interventions could reduce fear of childbirth and promote self-efficacy [41]. Another study by Carlsson et al. [42] found that self-efficacy correlated with reduced use of epidural analgesia among primiparous women, which may reflect their ability to exert control, and experience safety as observed in the present study.
In this study, fear of forthcoming births was significantly lower in both intervention groups compared to the control group. By using pairwise testing, we were able to control individual differences and obtain a clearer understanding of the impact of the intervention on participants' fear of childbirth. Klabbers et al. [43] showed in an RCT that haptotherapy could significantly reduce fear of birth compared to psycho-education via the internet and usual care. Haptotherapy is designed to promote a more positive attitude in pregnant women and change cognitive appraisal to improve readiness for childbirth [44]. The Birth APP, tested in this study, is based on the Birth Without Fear© method, which also focuses on cognitive aspects, aiming to strengthen both physical and emotional capacity [15]. Other studies have shown the importance of tested and valid apps for pregnant women, especially for women with anxiety during pregnancy [20] and emphasizes mHealth apps to align with pregnant women's preferences for a mHealth app during pregnancy [45, 46].

Other information

23) CONSORT: Registration number and name of trial registry

Please see the inserted answer above.

24) CONSORT: Where the full trial protocol can be accessed, if available

No harms were identified during the process. The intervention was totally voluntary.

25) CONSORT: Sources of funding and other support (such as supply of drugs), role of funders

Like this:ClinicalTrials.gov (ref. no NCT05122390), and the first version of the protocol was uploaded on November 16, 2021 No deviations from the registered protocol occurred.

X26-i) Comment on ethics committee approval

x26-ii) Outline informed consent procedures

Like this: This study was approved by the Swedish Ethical Board (ref.no 2021-03028) and registered in ClinicalTrials.gov (ref. no NCT05122390), and the first version of the protocol was uploaded on November 16, 2021 No deviations from the registered protocol occurred. All participating women provided informed consent via RedCap, after receiving study information and information about GDPR. To ensure confidentiality of the participants, all registered names and e-mails were replaced by a system-generated number provided in RedCap. This number was used to integrate their individual codes in the app, and data from the app was transferred to the researcher via a secure portal at Karlstad University, Sunet Drive. Participants completing the follow-up questionnaire received a gift card valued at SEK 200 for use in a supermarket. No potential risks to participants were identified in this trial.

X26-iii) Safety and security procedures

Like this: All participating women provided informed consent via RedCap, after receiving study information and information about GDPR.

X27-i) State the relation of the study team towards the system being evaluated
